# The Institutional Worker

**Published:** 1918-08-24

**Authors:** 


THE
### INSTITUTIONAL WORKER
Being a Special Supplement to "The Hospital"

### OUR BUREAU OF INFORMATION.

### Rules for Correspondents.

1. Every letter must be accompanied by the coupon to be cut from the back cover (inside page) of THE HOSPITAL, current issue, and must contain the name and address of the correspondent with pseudonym for publication if desired. Replies by post cannot be given save under exceptional circumstances at the Editor's discretion.
2. Letters from Approved Homes in reply to special needs published in the Bureau should state terms and full particulars, and be sent prepaid under cover to the Editor of the Bureau with name written across coupon for identification.
3. Proprietors of Homes which have not yet been entered on the List of Approved Homes, but have spare accommodation likely to suit special needs, are invited to write for an application form for registration. The fee for registration, which includes two announcements of the Home in the Bureau and other privileges, is 10s.
4. All communications to be addressed to the Editor of THE HOSPITAL, 28 Southampton Street, Strand, London, W.C. 2, and marked "Bureau of Information."

### INSTITUTION FACTS AND FIGURES.

### Bedroom Suppers for Nurses Off Duty.

#### Answer to July Question.

THE question was:

**In those hospitals having no kitchen in the Nurses' Home what arrangements can be made whereby the sisters and nurses who are off duty in the evening may have the comfort of supper in their bedrooms? What method may be adopted in order to avoid the dining-room maid being faced by a more or less depleted pantry when she wishes to lay breakfast? (The difficulty seems to be not so much as regards the food as the crockery.)**

**By "Scottie."**

Where there is no kitchen in the Nurses' Home special crockery and cutlery should be provided of a different pattern from that used in the dining-room; also Japanese trays, as they do not require tray-cloths; and rustless knives, thus saving laundry and knife polishing. A small gas-ring or steam-oven might be placed in the pantry, or, if that were not convenient, it might be possible to use a corner of a corridor for this purpose. Soup, milk, or cocoa might be left there to keep hot, and cups placed on a table or shelf so that nurses could help themselves to what they wanted. A tray could be left in the bedroom with biscuits and cheese or any food not requiring to be kept hot. A slate should be kept in a prominent place (as nurses are forgetful sometimes), on which those who require supper would write their names. The nurses would be expected to return trays, etc., to pantry or corridor before going on duty. The "washing up" should be done by the maid at the earliest possible moment, and the articles counted and placed in a cupboard with a lock so that nothing would be missing at stock-taking, which should be at frequent intervals.

**By "Provincial Matron."**

In order that sisters and nurses off duty may enjoy the comfort of suppers in their own rooms, and at the same time that the dining-room crockery may be kept intact, a few definite rules must be made and strictly kept. That nurses are very welcome to their suppers should be made clear by providing a sufficient supply of food to allow of some being taken away, and holding someone responsible for serving it. Allow no crockery from the dining-room to be borrowed or lent, and use a pattern that is not provided for any other department of the institution; also have it counted regularly. Advise each nurse to provide herself with a small tray and the necessary plates, and to bring or send these to the house sister when requiring a supper. This will not be looked upon as any hardship, while the freedom from responsibility which using her own crockery gives will add to the enjoyment of the supper. If an occasional supper-party be allowed, the fact that each member is asked to bring her own crockery, and the variety of china, will constitute part of the fun.

### Butter-Cutting Machines.

ST. BARTHOLOMEW'S HOSPITAL, London, uses an excellent device for cutting about four hundred 5-oz. butter rations every week, which results in absolute accuracy in weight and a great saving in labour previously expended in weighing each portion. This machine cuts and moulds each ration very quickly. The mechanism has an adjustment for altering the weight if required, but once set it will quickly turn out any number of portions of butter, margarine, lard, or similar substances exactly the same in weight and size. The outfit is nickel-plated, quite simple in construction, and can be taken to pieces in a few moments for the purpose of cleaning. Application has been made for a patent for this machine by Mr. C. R. Pyle, Clerk of the Works, St. Bartholomew's Hospital, West Smithfield, E.C. 1, who will be pleased to answer any inquiries respecting this machine.

**Economical Pudding.**

As you say, it is very difficult in these days to provide many changes in the nurses' diet, particularly puddings. You may find the following a useful recipe: Rub ¼ lb. of dripping with 6 oz of flour, two teaspoonfuls of baking powder, and 2 oz. sugar. Add one egg and sufficient milk to mix, and bake in the oven for about thirty minutes. Turn out and serve with a little jam on the top. Egg-powder will do in place of the egg. The quantity required would be two teaspoonfuls of egg-powder to two or three tablespoonfuls of milk.—DORIS.

### AUGUST QUESTIONS.

### 1. War Vacancies.

**Give model "Terms of Appointment" applicable for the substitute of a hospital's administrative official who has been called to the Colours. Provision to be made for the possible return to duty of the official previous to the termination of the war and the filling of the appointment in case of the non-return of the official concerned. Describe fully any experiences in this connection that may prove of value to hospital managers generally.**

### 2. Bedroom Suppers for Nurses Off Duty.

**In those hospitals having no kitchen in the Nurses' Home what arrangements can be made whereby the sisters and nurses who are off duty in the evening may have the comfort of supper in their bedrooms? What method may be adopted in order to avoid the dining-room maid being faced by a more or less depleted pantry when she wishes to lay breakfast? (The difficulty seems to be not so much as regards the food as the crockery.)**

### ENQUIRIES AND ANSWERS.

*THE SICK AND IN NEED.*

### Home for Mild Mental Case.

PROBABLY, if the patient's means permit, the best course is to arrange for care in a private home where mild mental cases are dealt with. Some of the better-known private asylums take uncertified as well as certified cases, as, for instance, St. Luke's Hospital for Mental Diseases, Old Street, London. At this institution the maximum charge (pre-war) was 30s. per week, though a great many cases are admitted gratuitously. If you would indicate whether the patient is male or female and the district in which a home is desired we could possibly give you more definite information.—M. I. B.

### Convalescent Homes Association.

THE address of the Convalescent Homes Association is 14 Victoria Street, London, S.W. The objects of this Association are to co-ordinate the work of Public Convalescent Homes receiving London convalescent patients and to obtain and disseminate information on the needs of London in respect of convalescent relief. The inquiry fee is 1s.—POPPY.

*MISCELLANEOUS.*

### Insurance Society.

WE can only suggest that you apply to the Royal National Pension Fund for Nurses, 15 Buckingham Street, Strand, W.C. 2, which has a sickness assurance branch.—ANXIOUS.

### The Dressings Shortage.

THE need for conserving hospital dressings, to which reference has been made in these columns, is universally recognised, but the desirability of reclaiming them is perhaps not generally admitted. Nevertheless some of the hospitals in this as well as other countries have been giving this matter their attention. The Canadian Laundry Machinery Company, Ltd., of Toronto, have provided a high-pressure sterilising washer which is utilised for this purpose. In this the soiled dressings are exposed to live steam at 320 degrees Fahrenheit under a pressure of 75 lbs. It is claimed that no known life can exist under these conditions for more than ten minutes.—CANADIAN.

